# Altered Hypothalamic functional connectivity in adolescents with severe obesity

**DOI:** 10.21203/rs.3.rs-8694593/v1

**Published:** 2026-02-02

**Authors:** Allison Shapiro, Meghan Pauley, Jaime M. Moore, Lucy Hall, Nicholas Stence, Susan Johnson, Kristen Nadeau, Kathleen Keller, Barbara Rolls, Marc-Andre Cornier, Jason Tregellas

**Affiliations:** University of Colorado Anschutz; University of Colorado Anschutz; University of Colorado School of Medicine; University of Colorado Anschutz; University of Colorado Anschutz; The Pennsylvania State University; The Pennsylvania State University; Medical University of South Carolina; University of Colorado, Anschutz Medical Campus

## Abstract

**Background/Objectives:**

Neurobiological frameworks of obesity in youth have focused largely on non-homeostatic systems (reward, salience, executive control), while the homeostatic system—particularly the hypothalamus—is comparatively understudied. A clearer picture of how these systems interact in adolescents with severe obesity is needed to inform treatment. This study sought to test whether adolescents with severe obesity exhibit altered hypothalamic functional connectivity, relative to healthy-weight peers, across fasting and fed states.

**Subjects/Methods:**

We analyzed data from the Food and Adolescent Brain Study, a single-blinded randomized cross-over trial (NCT04208256) of 13–18-year-old adolescents with severe obesity (SO; body mass index [BMI] >99^th^ %ile; n=30; mean [SD] age 14.6 years [1.5]) and with healthy weight (HW; BMI <85^th^ %ile; n=26; 15.5 years [1.6]).

**Interventions/Methods:**

Participants completed resting-state functional magnetic resonance scans during fasting and after ingesting a 75-gram glucose drink (1 255.8 kJ [300 kcal]) to induce a fed state. Multivariate general linear models were run in seed-to-voxel analyses to estimate functional connectivity, setting the hypothalamus as the seed region. All models were adjusted for age and sex, with significance determined via cluster-forming voxel-level p<0.005 and false discovery rate-corrected cluster-level p<0.05.

**Results:**

In adolescents with SO, during fasting, hypothalamic connectivity in adolescents with SO was weaker to the bilateral cerebellum and left(L) middle occipital gyrus, and stronger to the right(R) postcentral/supramarginal gyri, compared to the HW group. During the fed state, hypothalamic connectivity increased to the bilateral middle frontal gyri and R putamen and decreased to the R caudate and L superior frontal gyrus in adolescents with SO, relative to the HW group.

**Conclusion:**

Severe obesity in adolescence is associated with altered communication between homeostatic (hypothalamus) and non-homeostatic brain structures, evident across fasting and fed states. Findings underscore the need to incorporate homeostatic circuitry into pediatric obesity frameworks.

## INTRODUCTION

Through substantial preclinical work and a growing number of studies in humans, the brain has been increasingly implicated in the etiology of obesity(1). Indeed, enough evidence from human neuroimaging studies has amassed for neurobiological frameworks of obesity to emerge. Multiple frameworks have been proposed, with the most predominant being: 1) the reward surfeit model of obesity, which posits that people with obesity have a heightened reward response to appetizing foods, thus leading to overconsumption of such foods in an effort to achieve the desired reward; and 2) the inhibitory control deficit theory of obesity, proposing that people with obesity experience dysregulation of inhibitory control related to food stimuli, and therefore demonstrate eating disinhibition (reviewed in (2)). Taken together, these theories of human obesity – and by extension, much of the evidence underlying them (reviewed in (3)) – center on dysregulated non-homeostatic neural systems that contribute to overconsumption.

A large body of work in animals has demonstrated that eating behavior within the context of obesity is driven by both non-homeostatic and homeostatic (e.g. hypothalamic appetite/satiety signaling, etc.) neural systems. The non-homeostatic and homeostatic systems underlying eating behavior do not function as isolated units but instead are integrated circuits that comprise multiple communicating brain regions. The hypothalamus is the key homeostatic regulator of appetite control,(4, 5) and together with non-homeostatic systems that underly cognitive control,(6–8) salience,(9) and reward(10–12) comprises a circuit of functionally connected structures that influence eating behaviors (reviewed in (3)).

Adults and youth with obesity show altered neuronal activity in non-homeostatic brain regions in response to viewing and tasting highly palatable foods,(13–17) with the degree of altered neuronal activity related to overeating.(18–20) Hypothalamic dysfunction has been observed in adults(21) and youth(22) with obesity, where the hypothalamic response to a caloric stimulus – glucose or a liquid meal – is attenuated, relative to healthy weight counterparts (reviewed in (23)). In adults with obesity, functional connectivity between the hypothalamus and non-homeostatic brain networks has been shown to also be altered,(24, 25) suggesting dual-system disruption of the non-homeostatic and homeostatic eating behavior circuitry. In adolescents with obesity, however, altered hypothalamic communication, or connectivity, with non-homeostatic brain regions and networks is understudied, leaving a critical gap in our understanding of the neural contributors to obesity development and persistence in youth, now affects approximately 20% of American adolescents(26), with severe obesity (BMI ≥ 99th percentile for age and sex) being the most rapidly growing weight category in youth(27).

In the modern landscape of obesity treatment, powerful, neuromodulating pharmacotherapies such as glucagon-like peptide-1 receptor agonists (GLP-1RA) – shown to alter both non-homeostatic response to food (e.g., reward) and homeostatic control of eating behaviors via appetite suppression in adults, and metabolic bariatric surgery, also shown to reverse hypothalamic dysfunction(28) – are now available for use in pediatrics, and recommended for adolescents with severe obesity.(29) Importantly, adolescence is a uniquely dynamic developmental period during which both homeostatic (30) and non-homeostatic (e.g., reward and cognitive control(31, 32)) neural systems are undergoing rapid change, making them potentially sensitive to neuromodulating interventions. Studies investigating the underlying non-homeostatic and homeostatic neurobiology of severe obesity in adolescents are, therefore, critically needed to build a comprehensive understanding of the baseline neural circuitry within which these available interventions may exert a neuromodulating effect.

The primary objective of the current study was to examine functional connectivity of the hypothalamus, as a primary homeostatic structure, in adolescents with severe obesity, relative to their peers with healthy weight. We hypothesized that connectivity between the hypothalamus and non-homeostatic brain regions underlying reward, salience, and executive control would be weaker in adolescents with severe obesity, compared to adolescents with healthy weight. We further explored the effect of severe obesity in adolescence on whole brain functional connectivity, setting no *a priori* hypotheses, but expecting that adolescents with severe obesity would demonstrate different whole brain connectivity, again compared to their peers with healthy weight.

## MATERIALS/SUBJECTS AND METHODS

### Design and Participants

Data for the current analysis were collected as part of the Food and ADOlescent Brain (ADOB) Study, a single-blinded randomized cross-over study (NCT04208256) investigating the response of the adolescent brain to energy loads and their relationship to eating behaviors. The ADOB study recruited male and female adolescents, 13–18 years-old, with severe obesity (SO; body mass index [BMI] > 99th %ile for age and sex) or with healthy weight (HW; BMI < 85th and ≥ 10th %ile for age and sex) from the Children’s Hospital Colorado Lifestyle Medicine Clinic and the surrounding communities and high schools.

**Figure 1:**
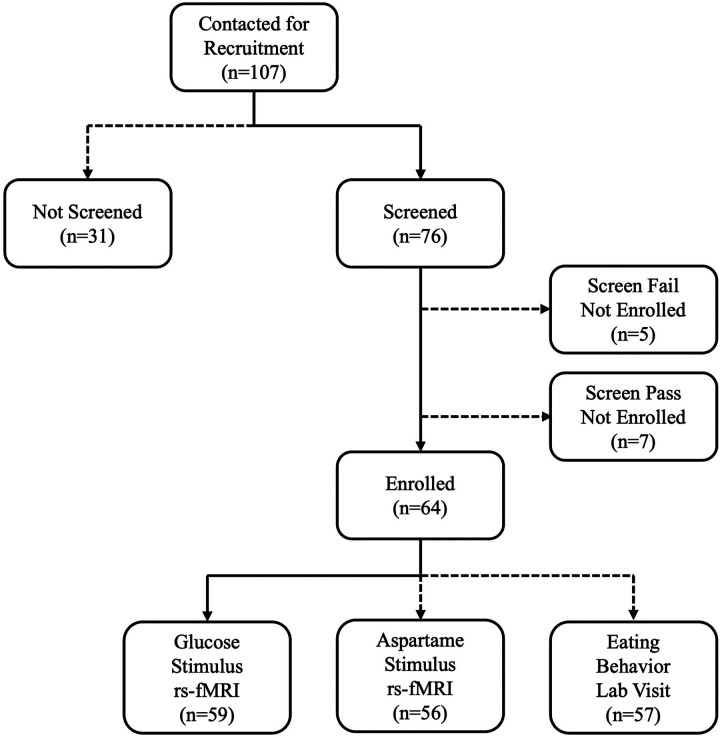
Consort diagram of study recruitment, enrollment, and visit completion. Solid lines represent participant data included in the current analysis. Dotted lines represent participant data not included in the current analysis.

Prospective participants were excluded from enrollment if they met any of the following criteria: prediabetes defined as a hemoglobin A1c ≥ 39 mmol/mol (5.7%) or physician’s diagnosis of diabetes; diagnosis of anorexia nervosa or bulimia nervosa; prescribed anti-psychotic medications (not including anti-depressant or anti-anxiety medications) or medications for weight loss or appetite suppression (e.g., phentermine, GLP-1RA); diagnosis of Chron’s or celiac disease or a serious food allergy (e.g., nuts); diagnosis of autism or Down’s syndrome, or any severe developmental disorder (excluding ADHD, dyslexia, or having an individual development plan in school); diagnosis of genetically-linked obesity or glioma in the hypothalamus or pituitary; a non-MRI safe device or metal in the body; claustrophobia. The ADOB study enrolled a total of 64 adolescents (SO = 34, HW = 30) between 2020 and 2024. See Fig. 1 for study consort diagram.

The ADOB protocol was approved by the Colorado Multiple Institutional Review Board (#19–1171). All participants 18 years of age provided written informed consent, with parents or guardians and participants providing written consent and assent, respectively, for those younger than 18 years of age.

### Data Collection

Eligible participants were invited to complete three research visits at the University of Colorado Anschutz. Research visits included two imaging visits and one visit during which participants completed observed, laboratory-based eating behavior assessments. Participants completed demographic and health questionnaires. Participants also completed the Pubertal Development Scale (PDS), a validated self-report questionnaire for pubertal staging in male and female adolescents(33). Five pubertal stage categories were derived from the PDS scores including pre-pubertal (male PDS score ≤ 3; female PDS score ≤ 2), early pubertal (male PDS score 4–5; female PDS score 2–3), mid-pubertal (male PDS score 6–8; female PDS score 4–6), late pubertal (male PDS score 9–11; female PDS score 7), and post-pubertal (male PDS score ≥ 12; female PDS score ≥ 8).

At enrollment, participants were randomized to receive an energy stimulus, equivalent to the fed state, in the form of an uncarbonated 300 ml orange-flavored 75-gram glucose drink (1 255.8 kJ [300 kcal]; Glucola^™^) at their first imaging visit, or a 300 ml energy neutral stimulus in the form of an uncarbonated orange-flavored aspartame sweetened drink mixture (Diet Orange Crush^™^). Whichever stimulus was not received at the first imaging visit was received at the second imaging visit (cross-over). In female participants, menstrual cycle was documented from self-report of date of last period and duration, and all visits completed during the luteal phase of their menstrual cycle to reduce potential variability in outcomes due to cycle-dependent fluctuations in sex hormones.

All scanning activities were conducted between 7 a.m. and 12 p.m. after an overnight fast at the University of Colorado Brain Imaging Center. Images were acquired on a Siemens 3T Skyra scanner (Siemens, Erlangen, Germany) with a 32-channel head coil and multi-slice acquisition (acceleration factor [SMS] = 3). Resting-state whole brain functional magnetic resonance imaging (rs-fMRI) data were acquired using the Blood Oxygen Level Dependent (BOLD) signal (TR = 1 s, TE = 35 ms, matrix 96 × 96, voxel size 3mm^3^). rs-fMRI scans were acquired in a restricted field of view (slices = 27) and by angling the field of view 15 degrees cephalic from the corpus callosum line with the most inferior slice placed just above the pituitary to reduce signal dropout in the hypothalamus from the sinus cavities. A T1-weighted anatomical scan (3D-MPRAGE, 192 slices, TR = 2 s, TE = 2.06 ms, matrix 256 × 256, voxel size 0.9mm^3^) was also collected. The participant’s head was stabilized within the coil using foam padding, and participants were instructed to keep their eyes closed and allow their mind to wander without falling asleep. During each imaging visit, a fasting rs-fMRI scan (600 volumes [~ 10 minutes]) was completed first, after which participants were slid out from the scanner bore and, with minimal body shifting, instructed to drink the entire volume of the given stimulus within a 5-minute period. The post-stimulus rs-fMRI scan (1 200 volumes [~ 20 minutes]) immediately followed the participant finishing the stimulus drink.

All enrolled participants (N = 64) completed at least one of the imaging visits. Per the primary aim of the study, as described in the protocol (NCT04208256), only data from the fed state imaging visit (fasting + post-stimulus with 75g glucose during) were used in the current analysis. Thus, participants were included if they completed the imaging visit with the 75-gram glucose stimulus (n = 59). From the 59 participants with fed state imaging data, 1 SO participant was excluded due to an anatomical anomaly, and 2 participants (1 SO, 1 HW) were excluded due to imaging quality control issues (> 10% of volumes displaying translation >1mm or rotation > 3 degrees). Thus, the analytic sample size for the current analysis was 56 adolescents (SO = 30, HW = 26; Fig. 1).

### fMRI Preprocessing and Analyses

All BOLD images were preprocessed in FSL using a standard pipeline including initial brain extraction via the Brain Extraction Tool (BET) and motion correction and 6-parameter estimation via the Flexible Linear Registration Tool (FLIRT). Spatial normalization was completed with the Flexible Non-Linear Registration Tool (FNIRT) to register BOLD and T1-weighted images to age-specific templates in Montreal Neurological Institute (MNI) standard space. Specifically, each participant was registered to the age-specific template closest to their age at the imaging visits (e.g., participant age = 15.4 y/o, registered to the 15.0 y/o template). Spatial smoothing with an 8mm full width at half max smoothing kernel was applied to the final registered image.

Connectivity analyses were completed using the CONN Toolbox in Matlab vR2024a. Preprocessed scans were denoised using a standard denoising pipeline including regressing out the WM timeseries (5 CompCor noise components), CSF timeseries (5 CompCor noise components), movement regressors and their first order derivatives (12 components), and linear trends within each functional run, followed by bandpass frequency filtering of the BOLD timeseries (0.008 Hz and 0.09 Hz).

### Hypothalamic Functional Connectivity

The hypothalamus ROI was defined by manual segmentation of the age-specific adolescent template(34) representing the median age of the full study sample (15.0 y/o). A seed-to-voxel analysis was run, in which the hypothalamus was set as the seed, and all voxels in the brain included as targets.

First-level, within-participant, seed-based and ROI-based connectivity maps (SBC) were estimated to characterize the patterns of functional connectivity between the hypothalamus and each voxel of the brain in the baseline/fasting and corresponding post-glucose load scan (2 conditions). Functional connectivity strength was estimated via bivariate regression coefficients from a weighted general linear model (GLM), defined separately for each seed(hypothalamus)-voxel pair, modeling the association between their BOLD signal timeseries.

A multivariate GLM was used in second-level (group) analyses to test, 1) the association between obesity status (SO vs. HW) and fasting hypothalamic connectivity, and 2) the association between obesity status and change in hypothalamic functional connectivity from fasting to fed states. All models were adjusted for sex (female vs. male) and household income (<$100 000 vs. ≥$100 000), as the SO and HW groups differed by these characteristics. Although previous studies have used self-identified race and ethnicity as covariates in models of brain-related outcomes, there is no biological reason to suspect different brain function outcomes by self-identified race and ethnicity. Thus, following the guidance of the American Academy of Pediatrics,(35) we did not adjust models for self-identified race and ethnicity.

In seed-to-voxel models, a separate GLM was estimated for each individual voxel, setting first-level connectivity measures at this voxel as the dependent variable (one independent sample per subject and one measurement per condition), and obesity status as the independent variable. Multivariate parametric statistics with random-effects across subjects and sample covariance estimation across multiple measurements were applied. Cluster-level inferences were based on parametric statistics from Gaussian Random Field theory. Results were thresholded using a combination of a cluster-forming p < 0.005 voxel-level threshold, and a familywise error corrected p-FDR < 0.05 cluster-size threshold.

### Untargeted Whole Brain Functional Connectivity

To explore the association between severe obesity in adolescence and whole brain connectivity, a voxel-to-voxel analysis was run with no *a priori* ROIs or seeds defined. First-level voxel-based connectivity maps were estimated to characterize the patterns of functional connectivity between each voxel in the brain. This was completed for both the baseline/fasting and post-glucose load scan. Following the same analytic approach as the above SBC analysis, in each participant, functional connectivity strength was estimated via bivariate regression coefficients from a weighted GLM, defined separately for each voxel-voxel pair, modeling the association between their BOLD signal timeseries. Multivariate GLM was again run, adjusted for sex (female vs. male) and household income (<$100 000 vs. ≥$100 000) to test, 1) the association between obesity status (SO vs. HW) and baseline/fasting whole brain connectivity, and 2) the association between obesity status and change in whole brain functional connectivity from baseline/fasting to post-glucose load. Given the exploratory nature of this analysis, we applied a two-sided test, and results were again thresholded using a combination of a cluster-forming p < 0.005 voxel-level threshold, and a familywise corrected p-FDR < 0.05 cluster-size threshold.

## RESULTS

Participants were on average 15 y/o [SD, 1.5], 55% had severe obesity, 52% were female sex at birth, and predominantly non-Hispanic White (65%). The SO and HW groups differed significantly by sex, race and ethnicity, and household income (p < 0.05 for all, respectively). Specifically, the SO group had a larger proportion of adolescents with male sex at birth (63%), adolescents who self-identified as Hispanic (27%) or non-Hispanic Black (10%), and adolescents from households with an annual income less than $100 000 (50%). By design, adolescents with SO had significantly higher BMI (p < 0.001), compared to adolescents with healthy weight. See Table 1 for participant characteristics reported by obesity status.

### Hypothalamic Functional Connectivity

**Figure 2:**
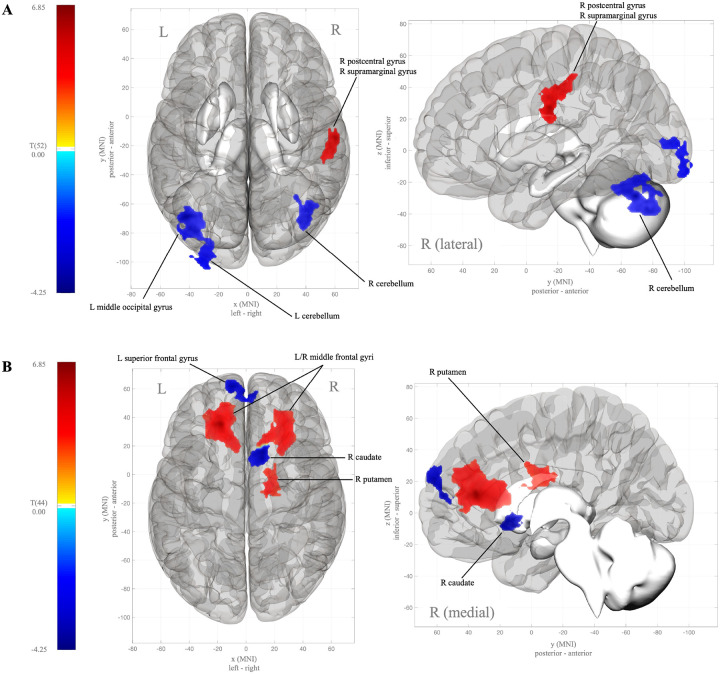
Hypothalamic connectivity in adolescents with severe obesity compared to adolescents with health weight during fasting (**A**) and during a fed state (post 75-gram glucose oral bolus) (**B**). Blue clusters indicate lower functional connectivity with the hypothalamus and red clusters indicate higher functional connectivity with the hypothalamus. R=right; L=left; MNI=Montreal Neurologic Institute standard coordinates.

Overall, independent of sex and age, hypothalamic functional connectivity during both the fasted and fed states was significantly different in adolescents with severe obesity, compared to their healthy weight counterparts (Table 2). Specifically, in the fasted state, hypothalamic connectivity to bilateral voxel clusters in the cerebellum and left middle occipital gyrus was significantly weaker among adolescents with severe obesity, compared to those with healthy weight (Fig. 2A). Compared to the healthy weight adolescents, those with severe obesity also showed significantly stronger hypothalamic connectivity to clusters in the right postcentral and supramarginal gyri during fasting.

After glucose ingestion in the fed state, adolescents with severe obesity showed a significant increase in hypothalamic connectivity to voxel clusters in the bilateral middle frontal gyri and right putamen (Fig. 2B), relative to the fasted state, and compared to their healthy weight counterparts. Further, in adolescents with severe obesity, hypothalamic connectivity to clusters in the right caudate and left superior frontal gyrus significantly weakened in the fed state, compared to the adolescents with healthy weight.

### Untargeted Whole Brain Functional Connectivity

**Figure 3:**
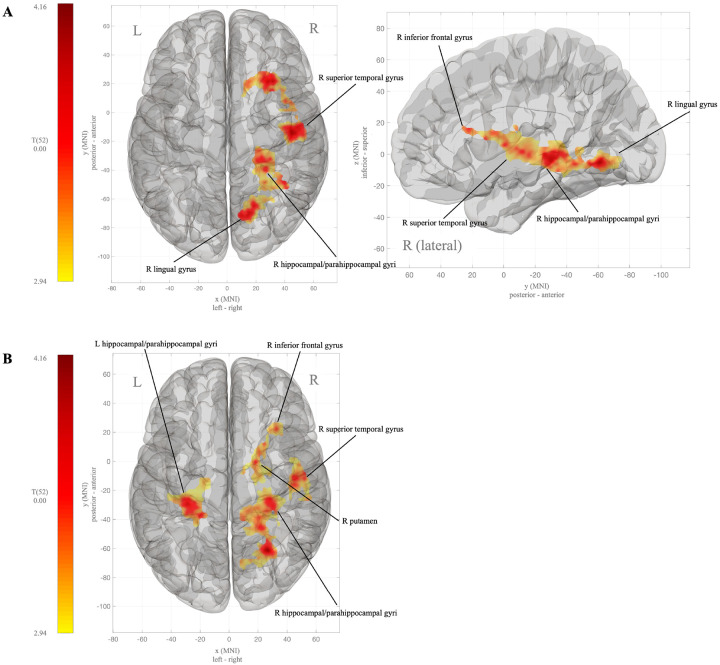
Whole brain connectivity in adolescents with severe obesity compared to adolescents with health weight during fasting (**A**) and during a fed state (post 75-gram glucose oral bolus) (**B**). Blue clusters indicate lower functional connectivity with the hypothalamus and red clusters indicate higher functional connectivity with the hypothalamus. R=right; L=left; MNI=Montreal Neurologic Institute standard coordinates.

Across all voxels in the brain during fasting, adolescents with severe obesity had significantly stronger whole brain functional connectivity with large voxel clusters exclusively within the right hemisphere that included the superior temporal and fusiform gyri, parahippocampal gyrus, and hippocampus, and in the right inferior frontal gyrus and lingual gyrus (Table 3; Fig. 3A), compared to those with healthy weight. In response to the glucose drink, relative to fasting, adolescents with severe obesity recruited left hemisphere structures such that, compared to those with healthy weight, they showed significantly increased whole brain functional connectivity with the bilateral parahippocampal gyri and hippocampus (Fig. 3B), as well as the right putamen, and fusiform and superior temporal gyri.

## DISCUSSION

We found that adolescents with severe obesity had significantly altered functional communication between the hypothalamus, a central homeostatic driver of appetite control, and other key homeostatic and non-homeostatic regions of the brain regions that are known to contribute to eating behaviors. To date, few published studies have investigated hypothalamic function or connectivity in children or adolescents with obesity, with most studies involving adults.(36, 37) Compared to the limited studies in children and youth, however, our results are broadly consistent.

In the fasted state, we observed hypoconnectivity between the hypothalamus and cerebellum among adolescents with severe obesity, relative to their healthy weight peers. Work over the past decade has revealed that the cerebellum, while traditionally thought to be primarily a motor control area, is also involved in eating behavior, where it contributes as an integration hub for food-related homeostatic and non-homeostatic (e.g., reward, affect) processes.(38) For example, in children without obesity, two recent studies have shown that loss of control eating, a form of eating disinhibition, is associated with stronger neuronal activation in the bilateral cerebellum in response to visual food cues that varied in portion size and energy density.(39, 40) While these studies were cross-sectional in design and did not include functional connectivity analyses, their results suggest that altered cerebellar responsiveness to food stimuli in late childhood may contribute to heightened risk for obesity via eating disinhibition.

Among adolescents with overweight or obesity (BMI ≥ 85th % ile), relative to adolescents with healthy weight, Martín-Pérez et al. (2018) found weaker resting state functional connectivity between the lateral hypothalamus and the right cerebellum, which is consistent with our fasted results.(41) Importantly, however, Martín-Pérez et al. conducted scanning after participants had consumed the main meal of the day, and thus, participants were assumed to be in a sated state during scanning. Despite clear methodological differences, the consistency between our results and those of Martín-Pérez et al. across energy states suggests that obesity in adolescence is related to energy state-independent disruption in signal integration between the brain’s primary homeostatic structures.

During the fed state, induced by oral glucose, a variable pattern of hypothalamic-corticostriatal connectivity emerged. Specifically, among adolescents with severe obesity, we observed stronger hypothalamic connectivity with regions implicated in reward-seeking behaviors (putamen(42)), and weaker hypothalamic connectivity with regions broadly involved in cognitive control and goal-directed behavior (superior frontal gyrus(43) and caudate(44)). These observed connectivity patterns partially align with the theory of heightened incentive salience of food cues among people with obesity, whereby cognitive control processes are disrupted or weakened and reward responsiveness and salience valuation are enhanced in response to visual, olfactory, or taste food cues (reviewed in(45)). We also observed stronger hypothalamic connectivity with the middle frontal gyrus (MFG) – a structure implicated in inhibitory control(46–48). While these results may appear to relate to the inhibitory control deficit theory of obesity and its supporting evidence, which shows lower *activation* of inhibitory control brain regions in response to food in people with obesity (reviewed in (49)), it remains unclear whether increased hypothalamus-to-MFG connectivity is indicative of an attenuated or compensatory inhibitory response to the glucose stimulus in the current study. Further work using response inhibition tasks, such as the go/no-go task, is needed to make such a distinction. The middle frontal gyrus is also implicated in working memory, thus, alternatively, the observed increased connectivity with the hypothalamus may represent attention allocation to glucose and/or sweet taste. (50)

Altogether, our hypothalamic connectivity results provide evidence of possible aberrant communication in, or integration of signaling across major homeostatic hubs and cognitive control and reward processes in adolescents with severe obesity, relative to their peers with healthy weight. In highlighting the potential significant role of disrupted homeostatic signaling in the neural circuitry present in obesity, our results suggest that in addition to reward processes that receive the majority of attention in models of pediatric obesity and obesogenic behaviors, it is likely important to also consider effects of homeostatic mechanisms that have been examined extensively in animal models of obesity (see (51) for an historical review). For example, preclinical models generated the roadmap of hypothalamic involvement in the pathogenesis of diet-induced obesity, specifically characterizing lateral hypothalamic nuclei and their functions in appetite regulation and energy homeostasis (reviewed in (52)), and demonstrating the mechanisms of neuropeptide signaling in the control of eating behaviors (reviewed in (53)). Additional research is needed to replicate our results and continue to understand involvement of homeostatic neural systems involvement in adolescent obesity. Moreover, it is critical for future research in the neural underpinnings of adolescent obesity to include investigation of homeostatic brain structures in addition to non-homeostatic circuitry.

In untargeted, whole brain analyses, functional connectivity differences across non-homeostatic structures predominated. We found state-independent increases in connectivity across regions underlying response inhibition (e.g., inferior frontal gyrus(54)), reward (e.g., putamen(42)), episodic and semantic memory (e.g., hippocampus(55)), and food perception (e.g., lingual gyrus(56)). These results may indicate sweet taste-elicited attentional bias(57) (“liking” response) and heightened incentive salience(58) (“wanting” response) in the adolescents with severe obesity. Thus, aligning with the current non-homeostatic theoretical models of obesity and confirming, in part, the presence of disrupted non-homeostatic neural processes in adolescents with severe obesity.

### Strengths and Limitations

Our results add novel insight into the potential homeostatic neural underpinnings of severe obesity in adolescents with a robust, physiologically informed study design that adds strength to our findings. Specifically, the imaging protocol included glucose ingestion as a stimulus for targeted engagement of the hypothalamus and an extended post-stimulus scan time (20 minutes) to improve BOLD signal detection and reduce imaging noise.(59) Of note, however, several design limitations remain. First and foremost, this study was cross-sectional and focused in adolescents who already had severe obesity, thus, we cannot infer whether or how the observed hypothalamic connectivity patterns contribute to development of severe obesity. Second, the hypothalamus responds to dietary macronutrients other than glucose, such as free fatty acids (FFA), which may elicit a different hypothalamic connectivity pattern when orally ingested. Thus, interpretation of our results is limited to hypothalamic response to a large glucose bolus, equivalent to drinking two 12 oz cans of regular soda. Third, given the oral route for glucose ingestion in our design, we cannot decipher whether the hypothalamic connectivity patterns observed were due to direct fuel sensing in the hypothalamus via circulating blood glucose levels or peripheral signaling to the hypothalamus via vagal nerve afferent pathways from the gut. Finally, we tested undirected synchronizations of the BOLD signal in our functional connectivity analyses, which do not provide information about the direction of connectivity between brain regions. This limits the interpretation of our results, such that we do not know whether, in response to a fed state, the hypothalamus was driving the signal to the observed functionally connected brain regions, or vice versa. Future work is therefore needed to investigate the directionality of functional communication between homeostatic and non-homeostatic neural systems.

### Conclusion

Our results broadly suggest possible disruption of the communication between homeostatic and non-homeostatic neural systems in adolescents with severe obesity. Considering dysfunction in both homeostatic and non-homeostatic systems as a component of the severe obesity phenotype may help to better inform our approach to obesity treatment in youth. Indeed, the American Academy of Pediatrics recently revised their clinical recommendations for pediatric obesity treatment to include use of anti-obesity medications and surgical options as first-line therapies alongside lifestyle interventions.(29) With the introduction of GLP-1RAs and bariatric surgery, both of which are shown to change hypothalamic function and salience and reward circuitry in adults with obesity(28, 60), the treatment landscape has itself expanded to include potent homeostatic and non-homeostatic neuromodulating approaches in obesity treatment. Thus, a dual systems approach to treating disrupted non-homeostatic *and* homeostatic neural connections alongside lifestyle therapy in adolescent obesity is now possible. However, further work in adolescents with severe obesity is needed to replicate our findings and to inform future clinical trials of neuromodulating treatments and brain function in youth with obesity to better understand the effect of such therapies have on modulating non-homeostatic and homeostatic brain function.

## Figures and Tables

**Table 1:** Characteristics of ADOB analytic sample by obesity status (N = 56).

	SO (n = 30)	HW (n = 26)	p-value^1^
Age (years), mean (SD)	14.6 (1.5)	15.5 (1.6)	0.07
Body mass index, mean (SD)	32.9 (5.3)	21.7 (2.4)	<0.001
Sex, n (%)			0.02
Female	11 (36.7)	18 (69.2)	
Male	19 (63.3)	8 (30.8)	
Pubertal Stage – Female^2^			0.50
Pre-pubertal, no menarche	1 (9.1)	0	
Early pubertal, no menarche	3 (27.3)	5 (27.8)	
Mid-pubertal, no menarche	7 (63.6)	13 (72.2)	
Late pubertal, menarche	0	0	
Post-pubertal, menarche	0	0	
Pubertal Stage – Male^2^			0.20
Pre-pubertal	18 (94.7)	6 (75.0)	
Early pubertal	1 (5.3)	2 (25.0)	
Mid-pubertal	0	0	
Late pubertal	0	0	
Post-pubertal	0	0	
Race or ethnicity, n (%)			0.02
Non-Hispanic White	18 (60.0)	18 (69.2)	
Non-Hispanic Black	3 (10.0)	0	
Non-Hispanic multiple race	1 (3.3)	6 (23.1)	
Hispanic	8 (26.7)	2 (7.7)	
Parent education, n (%)			0.06
High school degree/GED and less	4 (13.8)	0	
Some college or technical/vocational school	7 (24.1)	2 (8.0)	
College or graduate degree	18 (62.1)	23 (92.0)	
Not reported	1	1	
Household income, n (%)			0.04
<$50,000	7 (24.1)	2 (7.7)	
$50,000–100,000	7 (24.1)	2 (7.7)	
>$100,000	15 (51.8)	22 (84.6)	
Not reported	1	1	

1: p-values for comparison between SO and HW derived by Fisher’s exact test for categorical variables and Student’s t-test for continuous variables.

2: Pubertal staging was self-reported via the Pubertal Development Scale

**Table 2:** Region names of significant voxel clusters found in seed-to-voxel hypothalamic functional connectivity and whole brain functional connectivity under fasting and fed (75g glucose) states in adolescents with SO vs. HW.

Condition	Region(s) Name^1^	Peak Voxel (MNI: x, y, z)	KE^2^	p-Uncorrected	pFDR-Corrected
Fasting	l. cerebellum (−)	−42,−68,−28	246	<0.0001	0.0009
	r. cerebellum (−)	42,−68,−20	146	0.0002	0.0151
	r. postcentral gyrus, r. supramarginal gyrus (+)	60,−14,28	121	0.0005	0.0277
	l. middle occipital gyrus(−)	−28,−102,−12	106	0.0010	0.0394
Fed vs. fasting	l. middle frontal gyrus (+)	−18,36,14	672	<0.0001	<0.0001
	r. middle frontal gyrus (+)	26,26,10	584	<0.0001	<0.0001
	r. caudate (−)	16,−6,28	123	0.0005	0.0223
	l. superior frontal gyrus(−)	−10,62,22	109	0.0009	0.0302
	r. putamen (+)	12,12,−8	98	0.0016	0.0391

1: l. indicates the cluster was found in the left hemisphere; r. indicates the cluster was found in the right hemisphere; (+) indicates stronger connectivity within the cluster region, (−) indicates weaker connectivity within the cluster region.

2: KE (cluster size) for identified cluster

**Table 3:** Region names of significant voxel clusters found in whole brain voxel-to-voxel functional connectivity under fasting and fed (75g glucose) states in adolescents with SO vs. HW.

Condition	Region(s) Name^1^	Peak Voxel (MNI: x, y, z)	KE^2^	p-Uncorrected	pFDR-Corrected
Fasting	r. superior temporal gyrus, r. fusiform gyrus, r. hippocampus (+)	44,−14,8	244	0.0001	0.0138
	r. fusiform gyrus, r. parahippocampal gyrus, r. hippocampus (+)	40,−48,−6	200	0.0003	0.0146
	r. inferior frontal gyrus (+)	32,22,16	201	0.0003	0.0146
	r. lingual gyrus (+)	18,−64,8	150	0.0012	0.0455
Fed vs. fasting	r. parahippocampal gyrus, r. fusiform gyrus, r. hippocampus (+)	26,−60,6	551	<0.0001	0.0003
	l. hippocampus, l. parahippocampal gyrus (+)	−30,−30,−4	310	0.0002	0.018
	r. putamen (+)	18,0,6	190	0.0021	0.0498
	r. superior temporal gyrus	52,−12,2	184	0.0024	0.0498

1: l. indicates the cluster was found in the left hemisphere; r. indicates the cluster was found in the right hemisphere; (+) indicates stronger connectivity within the cluster region, (−) indicates weaker connectivity within the cluster region.

2: KE (cluster size) for identified cluster.
